# Cystic Fibrosis-Niche Adaptation of *Pseudomonas aeruginosa* Reduces Virulence in Multiple Infection Hosts

**DOI:** 10.1371/journal.pone.0035648

**Published:** 2012-04-25

**Authors:** Nicola Ivan Lorè, Cristina Cigana, Ida De Fino, Camilla Riva, Mario Juhas, Stephan Schwager, Leo Eberl, Alessandra Bragonzi

**Affiliations:** 1 Infection and Cystic Fibrosis Unit, Division of Immunology, Transplantation and Infectious Diseases, San Raffaele Scientific Institute, Milan, Italy; 2 Department of Microbiology, Institute of Plant Biology, University of Zurich, Zurich, Switzerland; Vrije Universiteit Brussel, Belgium

## Abstract

The opportunistic pathogen *Pseudomonas aeruginosa* is able to thrive in diverse ecological niches and to cause serious human infection. *P. aeruginosa* environmental strains are producing various virulence factors that are required for establishing acute infections in several host organisms; however, the *P. aeruginosa* phenotypic variants favour long-term persistence in the cystic fibrosis (CF) airways. Whether *P. aeruginosa* strains, which have adapted to the CF-niche, have lost their competitive fitness in the other environment remains to be investigated. In this paper, three *P. aeruginosa* clonal lineages, including early strains isolated at the onset of infection, and late strains, isolated after several years of chronic lung infection from patients with CF, were analysed in multi-host model systems of acute infection. *P. aeruginosa* early isolates caused lethality in the three non-mammalian hosts, namely *Caenorhabditis elegans*, *Galleria mellonella*, and *Drosophila melanogaster*, while late adapted clonal isolates were attenuated in acute virulence. When two different mouse genetic background strains, namely C57Bl/6NCrl and Balb/cAnNCrl, were used as acute infection models, early *P. aeruginosa* CF isolates were lethal, while late isolates exhibited reduced or abolished acute virulence. Severe histopathological lesions, including high leukocytes recruitment and bacterial load, were detected in the lungs of mice infected with *P. aeruginosa* CF early isolates, while late isolates were progressively cleared. In addition, systemic bacterial spread and invasion of epithelial cells, which were detected for *P. aeruginosa* CF early strains, were not observed with late strains. Our findings indicate that niche-specific selection in *P. aeruginosa* reduced its ability to cause acute infections across a broad range of hosts while maintaining the capacity for chronic infection in the CF host.

## Introduction

*Pseudomonas aeruginosa* is a common bacterium found in a wide range of environments; it infects nematodes, insects, plants, and ameba in the laboratory and probably encounters a similar range of potential hosts in the wild [1]. In humans, *P. aeruginosa* causes a wide range of infections, including deadly pneumonia when infecting immuno-compromised or cystic fibrosis (CF) patients. The clinical outcome of *P. aeruginosa* infection ranges from acute to chronic infections. Individuals in intensive care units can develop ventilator-associated pneumonia and/or sepsis as a result of *P. aeruginosa* infection. Patients with CF develop life-long chronic lung *P. aeruginosa* infection which leads to death.

Genomes of different *P. aeruginosa* isolates share a remarkable amount of sequence similarity when isolated from the environment or from different clinical origins [2], [3]. A considerable conservation of genes including nearly all known virulence factors, such as pyocyanin, a type III secretion system (T3SS), several proteases, lipases and phospholipases and rhamnolipids was observed in *P. aeruginosa* strains isolated from the environment, immuno-compromised patients and CF patients at the onset of infection [3]. Despite the overall genome similarity among diverse *P. aeruginosa* strains, point mutations accumulate in bacterial lineages persisting in CF airways. Mutations commonly acquired by *P. aeruginosa* strains during CF chronic infection are those in the regulators of alginate biosynthesis [4] and virulence genes involved in the LPS modification [5], motility [6], in the quorum-sensing regulation [7], [8], in biosynthesis of the T3SS [9] and multidrug-efflux pumps, and in mutator genes [10]. Changes in metabolic functions have also been described [11]. In addition, whole genome sequence analysis of *P. aeruginosa* longitudinal strains from the same CF patient revealed that a surprisingly large number of genes in the genome can be targets for mutation during adaptation to CF airways, although only a few of these genes were found to be affected in many of the late isolates [11]. Recent work demonstrated that the greatest contribution to the extremely high levels of genetic diversity is within an individual patient rather than between patients [12].

Pathogenicity of *P. aeruginosa* isolates from different habitats and clinical origin, including complex phenotypes from CF patients, can be strikingly different. Previous studies in the *P. aeruginosa* reference strains PA14 and PAO1, and additional strains from various sources, showed that the genes required for pathogenicity in one strain are neither required for nor predictive of virulence in other strains [2]. When comparing *P. aeruginosa* strains derived from the same type of infection, there was no consistent clustering with respect to their phenotype in *C. elegans* [2]. For example, urinary tract infection strains exhibited a wide range of virulent and avirulent phenotypes in acute infection models. In the same vein, both the most and the least virulent strains tested were isolates from CF infections. Taken together, these results suggest that virulence and in particular pathogenicity-related genes in different organisms are both multifactorial and combinatorial, and that the outcome of a specific host-pathogen interaction depends on the bacterial origin as well as on the host genetic background. Recent whole genome sequence analyses of *P. aeruginosa* strains isolated from CF patients described loss-of-function mutations in virulence genes, suggesting attenuation of virulence for CF-adapted strains [13]. In the case of CF infections, *P. aeruginosa* clonal populations remain isolated in a defined environment over a long period of time and normally do not spread to other patients. Whether *P. aeruginosa* phenotypes that have adapted to the CF-niches, have lost their competitive fitness in other environment is not known.

To expand our knowledge on *P. aeruginosa* virulence and how this bacterium interacts with its host, we tested the hypothesis that CF-niche adaptation and specialization reduces the bacterial pathogenic potential of the organism in acute infection models. We selected well characterized *P. aeruginosa* clonal lineages of strains isolated from three CF patients at the onset of infections and after several years of chronic colonization; the samples included late adapted strains carrying several phenotypic changes in virulence factor production, structural modification in the Pathogen-Associated Molecular Patterns (PAMPs) [14], [5], and patho-adaptive mutations within the genome temporally associated with CF lung infection [15]. These *P. aeruginosa* clonal lineages were tested in a multiple infection hosts, including *Caenorhabditis elegans*, *Galleria mellonella*, *Drosophila melanogaster* and mice with two different genetic backgrounds, C57bl/6NCrl and Balb/cAnNCrl, described previously as susceptible and resistant [16]. We showed that *P. aeruginosa* early strains were lethal in the multi-host models included in this study while late strains reduced or abolished acute virulence. Our findings suggest that the adaptation of different *P. aeruginosa* lineages within CF lungs selects populations with reduced pathogenic potential in acute infections which is maintained across a broad range of hosts.

## Results

### Pathogenic potential of *P. aeruginosa* sequential strains from CF patients in *C. elegans*, *D. melanogaster* and *G. mellonella*

**Figure 1:**
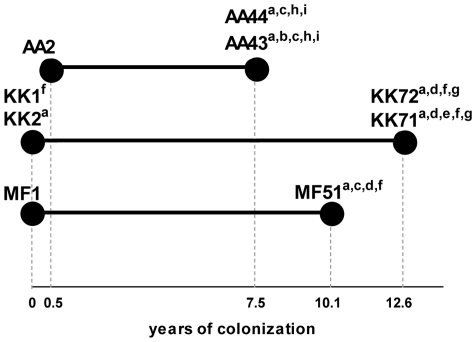
Genotypic and phenotypic characteristic of *P. aeruginosa* sequential isolates from CF patients. Three clonal lineages (AA, KK and MF) of *P. aeruginosa* strains were isolated at the onset of chronic colonization (early: AA2, KK1, KK2, MF1) or several years after acquisition and before patient's death (late: AA43, AA44, KK71, KK72, MF51). Clonality of strains was assessed by Pulsed Field Gel Electrophoresis and was reported previously [4]. Multiple phenotypic traits changed during genetic adaptation to the CF lung and included [14]: (a) motility defect, (b) mucoid phenotype, (c) protease reduction, (d) siderophore reduction, (e) hemolysis reduction, (f) LasR phenotype, (g) growth rate reduction. In addition, lipopolysaccharide (LPS) lipid A (h) and peptidoglycan (PGN) muropeptides (i) were analysed exclusively in the lineage AA showing specific structural modifications temporally associated with CF lung infection as described previously [5]. Additional data were reported in the online data supplement (**Table S1**).

**Figure 2:**
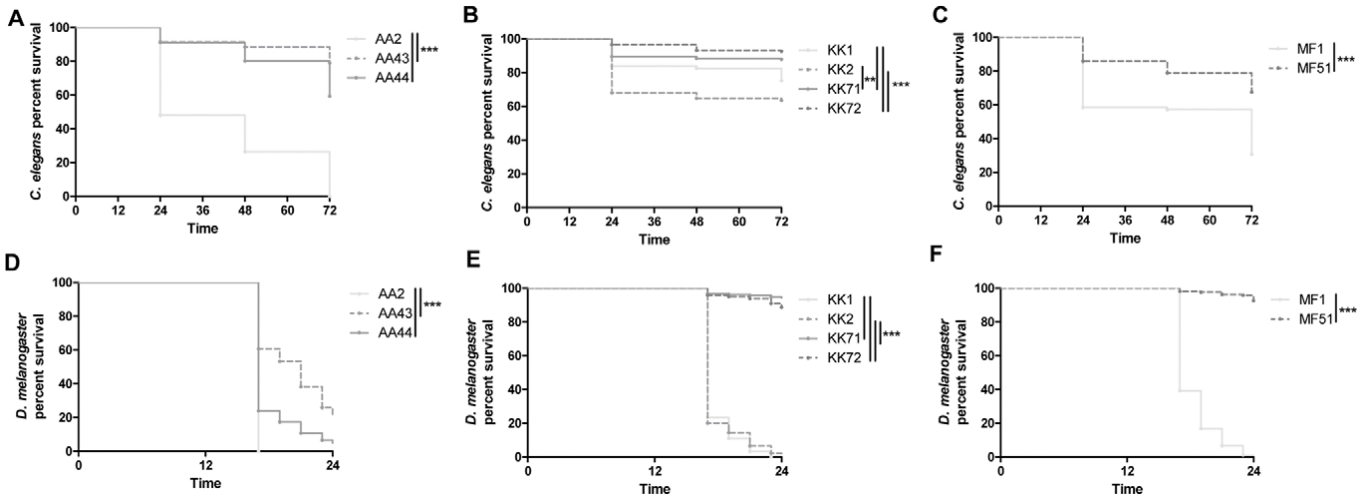
Pathogenicity of clonal pair of early/late *P. aeruginosa* isolates in *C. elegans* and *D. melanogaster*. Pathogenicity of lineages of early and late *P. aeruginosa* isolates in *C. elegans*: AA, (**A**); KK, (**B**); MF, (**C**); Pathogenicity of different lineages of early and late *P. aeruginosa* isolates in *D. melanogaster*: AA lineage, (**D**); KK lineage, (**E**); MF lineage, (**F**). Three independent experiments were pooled. Statistical analysis was calculated for pair wise comparisons between early and late strains (** p<0.01, *** p<0.001, Mantel-Cox test).

*P. aeruginosa* longitudinal strains isolated from three CF patients at the onset of infection (early) and after several years of chronic colonization (late) and carrying several phenotypic differences (**Figure 1** **and Table S1**) were tested for their virulence potential in three non-mammalian hosts, namely *C. elegans*, *D. melanogaster* and *G. mellonella*. In these experiments early *P. aeruginosa* strains, AA2, KK1, KK2 and MF1, and late strains, AA43, AA44, KK71, KK72 and MF51 were administered to non-mammalian hosts and mortality was monitored. *P. aeruginosa* early strain AA2 was significantly more lethal than the clonal late isolates AA43 and AA44 in *C. elegans* (AA2: 100% vs AA43: 21% and AA44: 41%, Mantel-Cox test: p<0.001) (**Fig. 2A**). Similar results were also obtained in *D. melanogaster* (AA2: 100% vs AA43: 82% and AA44: 97% p<0.001)(**Fig. 2D**). Although the late strains AA43 and AA44 were more pathogenic in this model in comparison to *C. elegans*, they killed the fruit flies later in comparison to the early strain AA2. Likewise, early *P. aeruginosa* isolates from patients KK and MF were significantly more lethal than their clonal late isolates in both models. However, lethality in *C. elegans* (KK1: 25% and KK2: 36% vs KK71: 11% and KK72: 7%, p<0.01; MF1: 69% vs MF51: 35%, p<0.001) (**Fig. 2B, C**) was generally less severe than in *D. melanogaster* (KK1: 100% and KK2: 100% vs KK71: 16% and KK72: 35%, p<0.01; MF1: 99% vs MF51: 11%, p<0.001) (**Fig. 2E, F**). We also evaluated lethality in a *G. mellonella* infection model. As in the previous models, the LD_50_ of the early isolate AA2 was found to be more than 20-folds reduced when compared to the clonal late isolates AA43 and AA44 (**Table 1**). Similar trends were also seen with the sets of early and late isolates (KK and MF isolates). The LD_50_ of the early isolates KK1 and KK2 were found to be more than 20-folds reduced when compared to the clonal late isolates KK71 and KK72. The early isolate MF1 showed a LD_50_ of 1500-folds reduced when compared to the clonal late isolate MF51, confirming the higher acute virulence of early strains.

**Table 1:** LD_50_ of longitudinal clonal *P. aeruginosa* lineages in *G. mellonella* larvae 24 hours post infection.

Strain	Nr. of Cells (LD_50_)
AA2	15
AA43	>3×10^2^
AA44	>3×10^2^
KK1	1.3×10^3^
KK2	2.8×10^4^
KK71	>6.0×10^5^
KK72	>7.0×10^5^
MF1	2.0×10^2^
MF51	>3.0×10^5^

>LD_50_ is higher than the maximum infection dosage used. Data represent mean values of at least three independent experiments.

### Response of different C57Bl/6NCrl and Balb/cAnNCrl inbred mouse strains to infection with *P. aeruginosa* sequential strains

**Figure 3:**
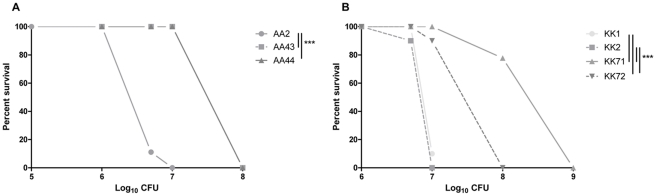
Correlation between survival percent and initial infection dose of clonal pair of early/late *P. aeruginosa* isolates in C57Bl/6NCrl. C57Bl/6NCrl mice were infected with different doses of *P. aeruginosa* strains from AA (**A**) and KK (**B**) clonal lineages. Survival of infected mice was followed over a period of 4 days and is indicated as a cumulative percent. Higher doses of late *P. aeruginosa* strains (AA43, AA44, KK71, KK72) are required for mortality when compared to early strains (AA2, KK1 and KK2). Two to three independent experiments were pooled (nr of mice: 5–18 as detailed in **table S2**). Statistical analysis of pair wise comparisons for early and late strains are indicated *** p<0.001 (Mantel-Cox test).

**Figure 4:**
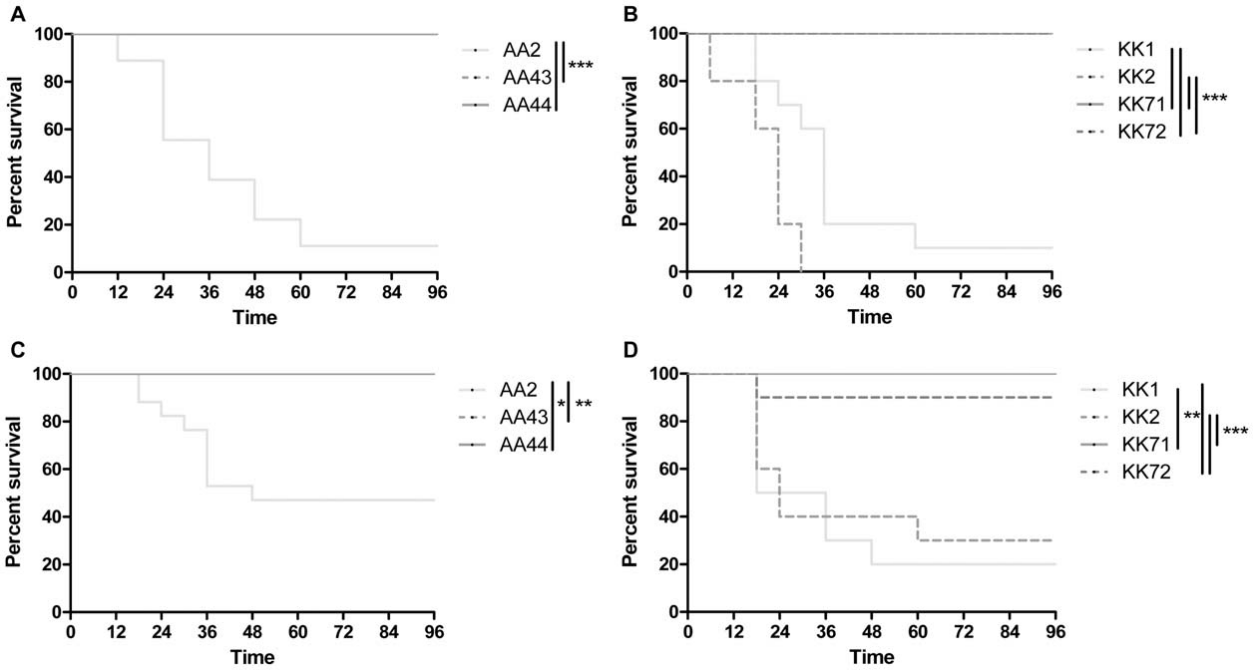
C57Bl/6NCrl and BALB/cAnNCrl inbred mouse strains exhibit a similar susceptibility after infection with clonal pair of early/late *P. aeruginosa* isolates. C57Bl/6NCrl (**A, B**) and BALB/cAnNCrl (**C, D**) mice were infected with 5×10^6^ cfu/lung of *P. aeruginosa* strains from AA (**A, C**) and 1×10^7^ cfu/lung KK (**B, D**) clonal lineages. Survival of infected mice was followed over a period of 4 days. Early strains (AA2, KK1 and KK2) were lethal while late strains (AA43, AA44, KK71, KK72) were attenuated in acute virulence. Two to three independent experiments were pooled (nr of mice: 5–18 as detailed in **table S3**). Statistical analysis was calculated for pair wise comparisons between early and late strains (* p<0.05; ** p<0.01; *** p<0.001, Mantel-Cox test).

To test whether the differences in lethality between early and late clonal *P. aeruginosa* strains are maintained in the mammalian host, we analyzed the host response in murine models of acute pneumonia. Lethality and changes in body weight in C57Bl/6NCrl and Balb/cAnCrl inbred mouse strains were assessed. First, escalating doses ranging from 10^5^ to 10^9^ cfu of *P. aeruginosa* were applied to C57Bl/6NCrl mice to determine the relative range of susceptibility. As shown in **Fig. 3** and **Table S2**, C57Bl/6NCrl died starting from 5×10^6^ cfu/lung of early AA2 strain and 1×10^7^ cfu/lung of early KK1 and KK2 strains, indicating differences in virulence between *P. aeruginosa* early strains of different lineages. When mice were inoculated at the same doses, late AA43, AA44, KK71 and KK72 strains were not lethal, indicating that their virulence was attenuated in comparison to the early strains (AA2 vs AA43 and AA44, p<0.001; KK1 and KK2 vs KK71 and KK72, Mantel-Cox, p<0.001) (**Fig. 4A and B**). In the AA lineage, all mice died at doses of 10^8^ cfu/lung of AA43 and AA44 strains (**Fig. 3A**), while in the KK lineage doses of 10^9^ cfu/lung of KK71 and 10^8^ cfu/lung of KK72 were fully lethal (**Fig. 3B**). Differences between early and late strains were also observed in BALB/cAnNCrl mice (AA2 vs AA43 and AA44, p<0.05; KK1 and KK2 vs KK71 and KK72, p<0.01) (**Fig. 4C and D**), which showed similar susceptibility as C57Bl/6NCrl. Bacterial cells were recovered from blood and other organs of moribund mice indicating that death was caused by sepsis (data not shown).

In accordance with these results, a major decrease in body weight was observed in mice infected with the early *P. aeruginosa* strains AA2, KK1 and KK2 when compared with the late clonal strains AA43, AA44 and KK71 both in C57Bl/6NCrl and Balb/cAnCrl (**Fig. S1**). Infections with KK72 strain appeared to be an exception.

### Histopathological lesions, localization and quantification of *P. aeruginosa* strains in the murine airways

**Figure 5:**
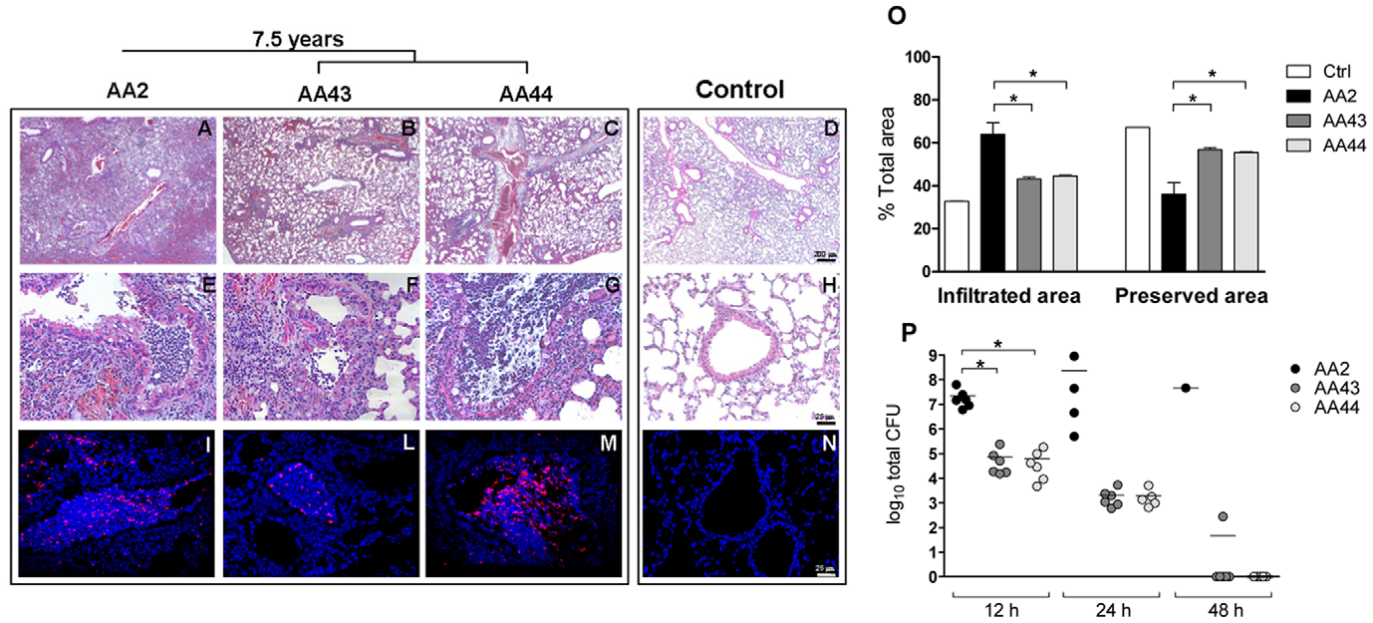
Histopathology, *P. aeruginosa* load and localization in murine lungs after infection with clonal pair of early/late *P. aeruginosa* isolates. C57Bl/6NCrl were infected with 5×10^6^ cfu/lung of *P. aeruginosa* strains from AA lineage for 24 hours. Control mice were not infected. Lungs were stained with H&E and in immunofluorescence with specific antibody against *P. aeruginosa* (red) (**A, E, I**: AA2; **B, F, L**: AA43; **C, G, M**: AA44; **D, H, N**: not infected). Counterstaining was performed with 4′,6-Diamidino-2-phenylindole dihydrochloride (DAPI) (blue). **I–N**) Bacterial cells of *P. aeruginosa* are visible in the bronchia and pulmonary parenchyma. O) Severity of lesions and lung involvement is heterogeneous in different lobes of the same mice. Quantification of infiltrated and preserved areas as percentage of total tissue area with mean ± SEM is shown (n = 3 mice each/strain). Statistical analysis was calculated for pair wise comparisons between early and late strains (* p<0.05; ** p<0.01; *** p<0.001, Mann–Whitney). **P**) Dots represent individual measurements of the no. of cfu per lung, and horizontal lines represent median values after 12, 24 and 48 h. Two independent experiments were pooled. Statistical analysis was calculated for pair wise comparisons between early and late strains (* p<0.05, Student's t-test).

To assess clinical strain-specific traits of acute pneumonia, lung histopathology was performed on mice challenged with strains of the *P. aeruginosa* AA clonal lineage for 24 hours. This analysis revealed that acute infection with early AA2 strain caused more severe lesions and leukocytes recruitment in the airways than infection with late AA43 and AA44 (**Fig. 5 A–C, E–G**). The area infiltrated with inflammatory cells was significantly increased in the AA2 strain compared to AA43 and AA44 infected mice (cell infiltration mean±SEM: 63.99±5.42% of AA2 vs 43.15±0.91% of AA43 and 44.51±0.44% of AA44, Mann Whitney test, p<0.05) (**Fig. 5O**). Accordingly, the percentage of tissue preservation was significantly higher for AA43 and AA44 compared to AA2 (36.01±5.42% of AA2 vs 56.85±0.91% of AA43 and 55.49±0.44% of AA44, p<0.05).

Immunofluorescence staining showed that the early strain AA2 was localized both within the bronchial lumen and within alveolar space (**Fig. 5I**), supporting its spreading to other organs during sepsis. In contrast, the late strains AA43 and AA44 were localized exclusively within the bronchia (**Fig. 5L, M**). Next, we quantified the bacterial load in the lungs of mice up to 48 h post infection. Given a starting dose of 5×10^6^, early AA2 strain replicated in the airways reaching a high load (2.3×10^8^ median CFU) and causing death of the animal (**Fig. 5P**). Late AA43 and AA44 decreased significantly bacterial numbers soon after infection and were completely cleared by the host immune system after 48 h, indicating a low pathogenic potential (AA2 vs AA43 and AA44, Student's t-test, p<0.05).

### Invasion of *P. aeruginosa* sequential strains in epithelial cells

**Figure 6:**
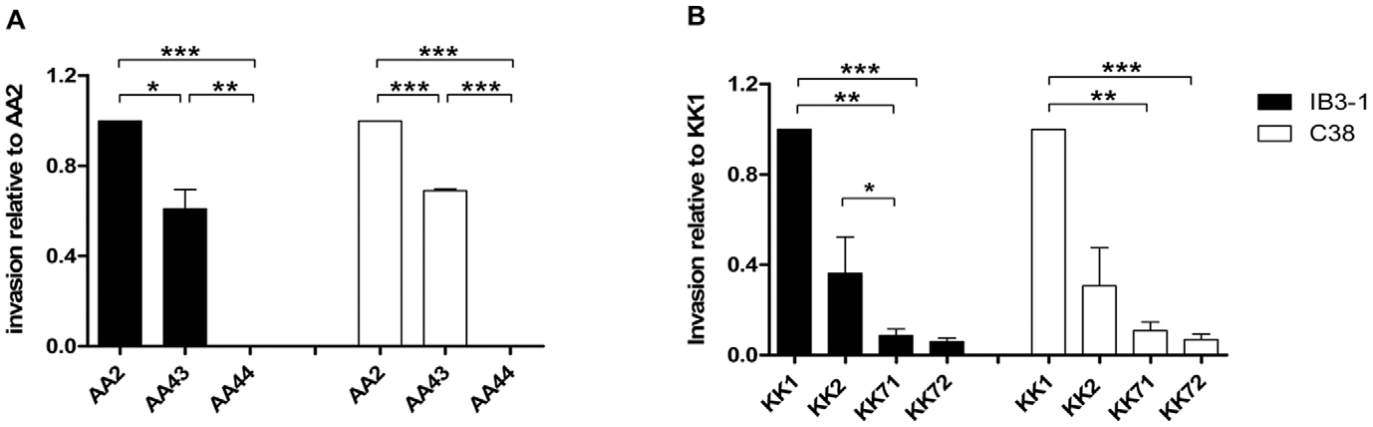
Invasion of clonal pair of early/late *P. aeruginosa* isolates in IB3-1 and C38 cells. **A**) Fold of invasion relative to AA2 after 1 h of stimulation with AA clonal lineage. **B**) Fold of invasion relative to KK1 after 1 h of stimulation with KK clonal lineage. Measurements were performed in triplicate. Statistical analysis was calculated for pair wise comparisons between early and late strains (* p<0.05, ** p<0.01, *** p<0.001, Student's t-test).

Bacterial invasion of host cells is a process common to many pathogens, including the CF-related pathogen, to evade extracellular immune factors [17] or to favour systemic spread [18]. We tested the ability of the *P. aeruginosa* clinical strains to invade CF respiratory cells (IB3-1) and isogenic corrected cells (C38). As shown in **Fig. 6**, early AA2 strain was found to be significantly more invasive than the *P. aeruginosa* clonal late strains AA43 and AA44 in both IB3-1 and C38 cells (IB3-1: AA2 vs AA43 p<0.05, AA2 vs AA44 p<0.001; C38: AA2 vs AA43 p<0.001, AA2 vs AA44, Student's t-test: p<0.001). In particular, AA44 strain was completely non-invasive in these experiments. Similar results were obtained with strains of the KK lineage. Early KK1 was significantly more invasive when compared to late KK71 and KK72 strains (IB3-1: KK1 vs KK71 and KK72, p<0.01 and p<0.001, respectively; C38: KK1 vs KK71 and KK72, p<0.01 and p<0.001, respectively). Early KK2 strain was more invasive than late clonal strains KK71 and KK72 both in IB3-1 and C38 cells, although significance was found only in IB3-1 cells (KK2 vs KK71, p<0.01).

## Discussion

Previous studies that were based on whole genome sequence analyses of longitudinal *P. aeruginosa* isolate from CF patients suggested that bacterial invasive functions are selected against during the course of chronic infection [5], [11]. Examples include motility, type III secretion system, O antigen biosynthesis, exotoxin, protease, and phenazine production, among others. Historically, many of these functions are considered to be virulence factors, as they provoke acute infection or dissemination within the host. Consequently, a major question that derived from previous reports and which requires further investigation was whether adaptation of *P. aeruginosa* strains to the CF-niche changes the fitness in other environments.

In this paper, we tested this hypothesis by evaluating acute pathogenicity of *P. aeruginosa* clonal variants from CF patients, in multiple infection hosts. The *P. aeruginosa* clonal strains included in this panel were isolated at different time points during CF chronic lung infection and were genetically characterized for genome rearrangements, mutations, and variations in pathogenic islands, and phenotypically for the loss of motility, acquisition of mucoidy, and a number of changes in the production of distinct virulence factors [14], [15]. Furthermore, the *P. aeruginosa* late strains, which were selected for this study, clustered with respect to their ability to persist in CF airways as well as in murine models of chronic infection that mimic the anaerobic conditions found the CF sputum [19], [14]. When *P. aeruginosa* longitudinal strains were tested in non-mammalian infection models, including *C. elegans*, *G. mellonella* and *D. melanogaster*, a reduction in acute virulence was observed in late strains relative to the respective early isolates. Most notably, there was a general agreement between the three models. When the panel of hosts was expanded to C57bl/6NCrl and Balb/cAnNCrl inbred mouse strains of different genetic backgrounds, we confirmed that early strains were lethal while late adapted *P. aeruginosa* strains were attenuated in acute virulence.

Based on previous reports on *P. aeruginosa* and other CF-related pathogens, this result was not obvious. In fact, the ability of bacteria to survive in a particular environment depends on virulence factors that are often specific for a particular host. Recent studies of *Burkholderia cenocepacia* infection in *C. elegans*, *G. mellonella*, alfalfa plant, mice and rats reported that most virulence factors are specific for one infection model only, and virulence factors are only rarely essential for full pathogenicity in multiple hosts. Only three factors were found to be essential for full pathogenicity in several host. *Burkholderia cenocepacia* mutants defective in quorum sensing, siderophore production and LPS biosynthesis were found to be attenuated in at least three of the infection models [20]. In *P. aeruginosa* PA14 strain only few host-specific virulence factors could be identified, and many of the mutants were attenuated in virulence in different hosts including *C. elegans*, *G. melonella* and mice [21]. However, when the virulence factors discovered in reference strains PA14 and PAO1 were tested in other clinical strains, no correlation between the absence and presence of these genes with virulence was observed [21]. Comparison of various clinical *P. aeruginosa* strains revealed that virulence is both multifactorial and combinatorial, the result of a pool of pathogenicity-related genes that interact in various combinations in different genetic background. *P. aeruginosa* clinical strains from the same type of infection exhibited a wide range of virulence in *C. elegans* [2]. For example, both the most and the least virulent strains tested were isolates from CF infections. Examination of specific genes among the several *P. aeruginosa* isolates did not reveal a consistent clustering of their genomic content with their pathogenic potential.

However, distinction between early and late *P. aeruginosa* strains from patients with CF had not been taken into account in previous works. Here, we directly compared *P. aeruginosa* early and late strains adapted to the CF-niche, which included strain exhibiting diverse phenotypes and belong to different genotypes, in several hosts. Although the genomes of *P. aeruginosa* isolates used in this work were not fully sequenced and only few phenotypic differences were identified, it is likely that late strains have accumulated several mutations during chronic persistence which account for the reduced pathogenicity across a broad range of hosts [14], [15]. Thus, the genetic adaptation process that leads to CF-niche specialization restricts the overall virulence of late strains to other environmental niche. Regarding the *P. aeruginosa* strains selected in this study, this process is not strain-dependent but is consistent for all the late isolates.

We were unable to correlate the observed differences in virulence of early and late *P. aeruginosa* strains with a specific phenotype, but it is most likely that multiple mutations are responsible for the attenuation of late strain in acute virulence. Notably, a single phenotypic difference of the early KK1 and KK2 strains, including a LasR phenotype and a motility defect, did not change virulence in several hosts; major pathogenic differences are evident in KK71 and KK72, in which multiple phenotypic changes were observed. The mucoid AA43 and the non-mucoid AA44 strains did not differ in their virulence potential. In addition, AA43 and AA44 were similarly attenuated despite their differences in LPS lipid A, as has been reported previously [5].

Although rodents are the first choice for understanding infectious diseases in human, non-mammalian models can be useful surrogate hosts. *Drosophila* response to pathogens and mammalian innate immune defenses are characterized by pathways conserved in vertebrates [22]. *C. elegans* has been largely used to identify virulence factors [23], allowing the study of responses to infection as well as comparison of the virulence of clinical and environmental isolates [24]. More recently both model organisms, *C. elegans* and *Drosophila*, have been also used to study host tolerance in addition to resistance mechanisms [25]. Their innate immune system employs evolutionary conserved signalling pathways [26], [21]. In reference to *G. mellonella*, it has been shown to correlate with mice models, when used to test *P. aeruginosa* virulence [27]. The use of non-mammalian infection models has several downsides, such as the specific temperature for the cultivation of the nematode may inhibit expression of certain virulence factors, the absence of the target organ and the lack of specific receptors or pathways. However, our results demonstrate the usefulness of these models for evaluating differences in acute virulence of *P. aeruginosa*.

Regarding the mammalian host, several studies have demonstrated that the host resistant/susceptibility response relies not only on the animal species but also on its genetic background [28], [29], [30], [16]. In particular, different susceptibility to *P. aeruginosa* chronic bronchopulmonary infection has been reported among genetically well-defined inbred mouse strains when mice were exposed to clinical strains embedded in the agar beads. Based on the bacterial load detected in the lung after three days and two weeks, Balb/cAnCrl mice were found to be resistant and C57Bl/6NCrl mice were identified as susceptible in two different studies [31], [28]. So far, direct comparison of the susceptibility of murine inbred strains to *P. aeruginosa* early and late strains from CF patients has not been performed. In our study, C57Bl/6NCrl and Balb/cAnCrl showed similar susceptibility to *P. aeruginosa* acute infection in terms of mortality but differences in pathogenicity among clonal early and late *P. aeruginosa* isolates observed in non-mammalian hosts. The genetic diversity of the mice in addition to the differences among type of infection (e.g. acute, reported in this work, and chronic, reported in previous works) and challenge, and bacterial origin may account for the different results [28], [29], [30], [16], [32]. Separate breeding colonies of C57Bl/6 mice maintained at the Charles River (“NCrl”), used in this study, or Jackson (J), used in previous studies, have led to the emergence of distinct substrains of C57Bl/6 mice that may explain the different susceptibility.

However, the findings that *P. aeruginosa* early strains were more lethal when compared to late strains in two different mouse genetic backgrounds strongly support the results in non-mammalian hosts that CF-niche adaptation of *P. aeruginosa* selects populations with reduced pathogenic potential in the acute infections. In addition, it has been argued that a high burden of infection but low virulence should account for host tolerance [33], [34], [25]. Consequently, our results indicated an increased host tolerance against *P. aeruginosa* CF adapted strains, as suggested by the high bacterial load sustained by the host. Our previous study showed that PAMPs of these *P. aeruginosa* strains, which were isolated at the late stage of CF chronic infection, drastically impair the host immune detection system suggesting a role of adaptation in increasing host tolerance [5], [35], [25]. Histopathological analysis carried out in this work supports the previous findings that detection of *P. aeruginosa* adaptive strains is impaired compared to early strains. The mechanism(s) that permits *P. aeruginosa* to cause invasive infections with bacteremia or tolerance is not known. Some bacterial pathogens can induce their own uptake into host cells (invasion), allowing the pathogen to enter a protected niche and, in some cases, to pass through cellular barriers including the respiratory epithelium and/or the blood barrier [17], [18]. Although further studies are needed to determine the exact mechanisms of *P. aeruginosa*/host interaction, it is tempting to speculate that the invasiveness of *P. aeruginosa* early strains may facilitate spreading from the lung to other tissues, while *P. aeruginosa* late strains, which are not able to protect themselves, may be finally eliminated.

Taken together, our results demonstrate that *P. aeruginosa* adaptation in CF airways selects pathoadaptive variants with a strongly reduced ability to cause acute infection processes in a host-independent way. These results have important implications for our understanding of the pathogenesis of *P. aeruginosa*-host interaction.

## Materials and Methods

### Ethics Statement

Animal studies were conducted according to protocols approved by the San Raffaele Scientific Institute (Milan, Italy) Institutional Animal Care and Use Committee (IACUC) and adhered strictly to the Italian Ministry of Health guidelines for the use and care of experimental animals.

Research on the bacterial isolates from the individuals with CF has been approved by the responsible physician at the CF center at Hannover Medical School, Germany. All patients gave informed consent before the sample collection. Approval for storing of biological materials was obtained by the Hannover Medical School, Germany.

### Bacterial strains and CF patient

Nine sequential *P. aeruginosa* isolates from three CF patient carrying ΔF508/ΔF508 or ΔF508/R553X *cftr* mutation were chosen from the strains collection of the CF clinic Medizinische Hochschule of Hannover, Germany. Genotypic and phenotypic data of *P. aeruginosa* strains were published previously and summarized in **Figure 1** and **Table S1** [4], [14], [5]. *P. aeruginosa* was cultured in *Pseudomonas* isolation agar (PIA) or Trypticase Soy Broth (TSB) at 37°C.

### Investigation of pathogenicity in the *C. elegans* model

For the investigation of pathogenicity *C. elegans* strain DH26 has been used. Worms were synchronized into L4 larval stage by egg preparation, which was followed by incubation of isolated eggs on *E. coli* OP50 feeding plates at 20°C for around 76 hours. Subsequently, L4 larvae were transferred on the lawns of examined bacterial strains grown in the 6-well plates (approximately 30 worms per well) and incubated at 25°C. The surviving worms were counted after 24, 48 and 72 hours with the aid of a Stemi SV 6 microscope (Zeiss, Goettingen). The pathogenicity of the investigated bacterial strains was determined from the survival rates of *C. elegans* in three independent replicates.

### *G. mellonella* killing assays

Infection of *G. mellonella* larvae was performed as described previously [27], with some modifications. Caterpillars in the final larval stage (Brumann, Zurich, Switzerland) were stored in wooden shavings at 15°C and used within 2 to 3 weeks. Bacterial overnight cultures grown in LB broth were diluted 1∶100 in 30 ml fresh medium and grown to an OD600 of 0.4 to 0.7. Cultures were centrifuged and the cellswere resuspended in 10 mM MgSO4 (E. Merck, Dietikon, Switzerland). 10-µl aliquots of three dilutions were injected into *G. mellonella* via the hindmost proleg using a 1-ml syringe (BD Plastipak, Madrid, Spain) with a 27-gauge needle (Rose GmbH, Trier, Germany).. Six healthy, randomly chosen larvae were injected and incubated at 30°C in the dark. As a control larvae were injected with 10 µl MgSO_4_. The number of dead larvae was scored 24 h after infection and the LD_50_ dosage was determined. Data are mean values for at least three independent experiments.

### Fly pathogenicity assay

Fly pricking assays were performed essentially as described by Apidianianakis *et al* [36]. 1 ml of an overnight culture was pelleted by centrifugation (10 min by 5000 rpm) and re-suspended in 1 ml of 10 mM MgSO_4_ solution. A Tungsten stainless steel needle, with approximate diameters of 0.01 mm at the tip and 0.2 mm across the main needle body, was dipped into the bacterial solution and pricked into the middle dorsolateral thorax of anesthetized flies. For each strain 15 flies were used. As a control, the flies were pricked with MgSO_4_ buffer. The infected flies were kept in glass vials, which were incubated at 26°C. Survival of the flies was monitored over time.

### Mouse model of acute *P. aeruginosa* infection

C57Bl/6 mice (20–22 gr) were purchased by Charles River. Mice were housed in filtered cages under specific-pathogen conditions and permitted unlimited access to food and water. Prior to animal experiments, the clinical *P. aeruginosa* strains were grown for 3 h to reach exponential phase. Next, the bacteria were pelleted by centrifugation (2700 g, 15 min), washed twice with sterile PBS and the OD of the bacterial suspension was adjusted by spectrophotometry at 600 nm. The intended number of cfu was extrapolated from a standard growth curve. Appropriate dilutions with sterile PBS were made to prepare the inoculum of 2×10^6^ up to 2×10^10^ cfu/ml. Mice were anesthetized and the trachea directly visualized by a ventral midline incision, exposed and intubated with a sterile, flexible 22-g cannula attached to a 1 ml syringe according to established procedures [14] [37]. A 50 µl inoculum of 1×10^5^ up to 1×10^9^ cfu were implanted via the cannula into the lung, with both lobes inoculated. After infection, mortality and body weight were monitored in one group of mice over one week. In another group of mice, the lungs were excised, used for histopathology, homogenized and plated onto TSB-agar plates for cfu counting.

### Histological examination and immunofluorescence

Mice were sacrificed by CO_2_ administration after 12, 24, 48 h of infection, lungs were removed en bloc and fixed in 10% buffered formalin at 4°C for 24 h, and processed for paraffin embedding. Longitudinal sections of 5 µm from the proximal, medial and distal lung regions were obtained at regular intervals using a microtome. Sections were stained with H&E according to standard procedures. Areas of inflammatory cell infiltration and tissue preservation (normal histology) were quantified using Image J software (National Institutes of Health) and reported as a percentage of total area [38]. Localization of *P. aeruginosa* was performed in de-paraffinized lung sections by employing a rabbit antiserum specific for *P. aeruginosa* and Texas Red-labeled goat anti-rabbit IgG as described [14]. The slides were examined using an Axioplan fluorescence microscope (Zeiss), and images were taken with a KS 300 imaging system (Kontron).

### Cell cultures and invasion assay

IB3-1 cells, an adeno-associated virus-transformed human bronchial epithelial cell line derived from a CF patient (ΔF508/W1282X) and C38 cells, the rescued cell line which expresses a plasmid encoding a copy of functional CFTR, were obtained from LGC Promochem [39]. Cells were grown in LHC-8 media (Biosource) supplemented with 5% fetal bovine serum (FBS) (Cambrex Bio Science). All culture flasks and plates were coated with a solution of LHC-basal medium (Biosource) containing 35 µg/mL bovine collagen (BD Biosciences), 1 µg/mL bovine serum albumin (BSA, Sigma) and 10 µg/mL human fibronectin (BD Bio Science) as described [40].

Bacteria invasion assay was performed using Polymyxins B (100 µg/ml) (Sigma) protection assay with minor modifications [41]. *P. aeruginosa* strains, grown to the mid-exponential phase, were used to infect cell monolayers at a 100∶1 multiplicity of infection for 1 h. The monolayers were washed with PBS, treated with antibiotic for 1 h, washed, lysed with H_2_O and plated on TSB-agar plates (Difco).

### Statistical analysis

Results are presented as median or mean±SEM. Student's t-test, Mann-Whitney test, Mantel-Cox test were used to determine the significance of differences in means between pairs.

## Supporting Information

Figure S1 **Weight change after infection with clonal pair of early/late** ***P. aeruginosa*** **isolates in C57Bl/6NCrl and BALB/cAnNCrl inbred mouse strains.** (**A**) C57Bl/6NCrl weights after infection with *P. aeruginosa* AA clonal lineage; (**B**) C57Bl/6NCrl weights after infection with *P. aeruginosa* KK clonal lineage; (**C**) BALB/cAnNCrl weights after infection with *P. aeruginosa* AA clonal lineage; (**D**) BALB/cAnNCrl weights after infection with *P. aeruginosa* KK clonal lineage. Data are expressed as mean ± SEM. Two to three independent experiments were pooled (nr of mice: 5–18 as detailed in table S3).
(TIF)

Table S1 **Genotypic and phenotypic characteristics of** ***P. aeruginosa*** **strains used in this work.**
(DOC)

Table S2 **Dose response in C57Bl/6NCrl infected with** ***P. aeruginosa*** **clonal lineages.**
(DOC)

Table S3 **Comparison between C57Bl/6NCrl and BALB/cAnCrl infected with** ***P. aeruginosa*** **clonal lineages.**
(DOC)
