# Supplementary figures and images for: Cystic Fibrosis-Niche Adaptation of Pseudomonas aeruginosa Reduces Virulence in Multiple Infection Hosts
Source: PLoS One. 2012 Apr 25;7(4):e35648. doi: 10.1371/journal.pone.0035648 (PMC3338451; doi:10.1371/journal.pone.0035648)

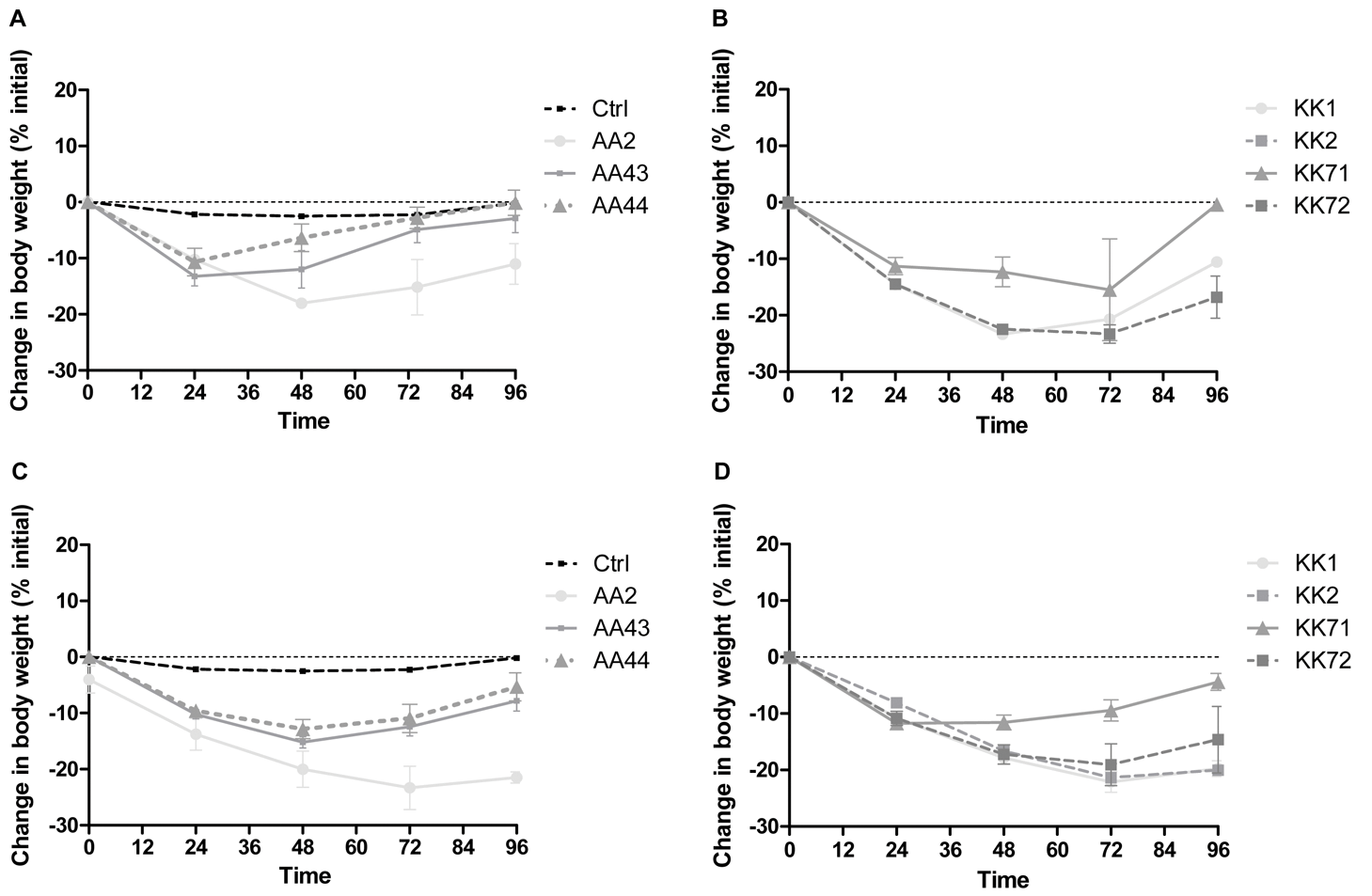

Supplement: Figure S1 — Weight change after infection with clonal pair of early/late P. aeruginosa isolates in C57Bl/6NCrl and BALB/cAnNCrl inbred mouse strains. (A) C57Bl/6NCrl weights after infection with P. aeruginosa AA clonal lineage; (B) C57Bl/6NCrl weights after infection with P. aeruginosa KK clonal lineage; (C) BALB/cAnNCrl weights after infection with P. aeruginosa AA clonal lineage; (D) BALB/cAnNCrl weights after infection with P. aeruginosa KK clonal lineage. Data are expressed as mean ± SEM. Two to three independent experiments were pooled (nr of mice: 5–18 as detailed in table S3). (TIF) [file pone.0035648.s001.tif]
