# Supplementary material for: Cystic Fibrosis-Niche Adaptation of Pseudomonas aeruginosa Reduces Virulence in Multiple Infection Hosts
Source: PLoS One. 2012 Apr 25;7(4):e35648. doi: 10.1371/journal.pone.0035648 (PMC3338451; doi:10.1371/journal.pone.0035648)
Supplement: Table S1 — Genotypic and phenotypic characteristics of P. aeruginosa strains used in this work. (DOC) [file pone.0035648.s002.doc]

**Cystic Fibrosis-niche adaptation of *Pseudomonas aeruginosa* reduces virulence in multiple infection hosts.**

Nicola Ivan Lorè, Cristina Cigana, Ida De Fino, Camilla Riva, Mario Juhas, Stephan Schwager, Leo Eberl, Alessandra Bragonzi.

Online Data Supplement

**Table S1**. **Genotypic and phenotypic characteristics of *P. aeruginosa* strains used in this work.**

| **Patient genotype** | **Strain** | **Origin** | **Years of colonization** | **Strain genotype**  **(PFGE)** | **Relevant phenotype** | **References** |
| --- | --- | --- | --- | --- | --- | --- |
|  |  |  |  |  |  |  |
| CF, ∆F508/∆F508 | AA2 | clinical | 0,5 | Θ | - | [4, 5, 14] |
|  | AA43 **^φ^** | clinical | 7,5 | Θ | Motility defect^∂^  Protease reduction  Mucoid  LPS and PGN changes^*^ | [4, 5, 14] |
|  | AA44 **^φ^** | clinical | 7,5 | Θ | Motility defective^∂^  Protease reduction  LPS and PGN changes^*^ | [4, 5, 14] |
|  |  |  |  |  |  |  |
| CF, ∆F508/∆F508 | KK1 | clinical | 0 | M1 | LasR phenotype ^τ^ | [4, 14] |
|  | KK2 **^φ^** | clinical | 0 | M1 | Motility defect^∂^ | [4, 14] |
|  | KK71 **^φ^** | clinical | 12,6 | M1^#^ | Motility defect^∂^,  Siderophore and hemolysis reduction  LasR phenotype ^τ^,  Growth rate reduction | [4, 14] |
|  | KK72 **^φ^** | clinical | 12,6 | M1^#^ | Motility defect^∂^,  Siderophore and hemolysis reduction  LasR phenotype ^τ^  Growth rate reduction | [4, 14] |
|  |  |  |  |  |  |  |
| CF, ∆F508/R553X | MF1 | clinical | 0 | K | - | [4, 14] |
|  | MF51 **^φ^** | clinical | 10,1 | K | Motility defect^∂^  Siderophore and protease reduced  LasR phenotype ^τ^ | [4, 14] |

^#^ Indicates genetic rearrangements within *P. aeruginosa* clonal lineage as assessed by PFGE.

^Φ^ Indicates the last *P. aeruginosa* strain prior to death or prior to lung transplantation

^∂^ Indicates swimming and twitching motility zone diameter, as measured by subsurface stab assay

^τ^ Isolates with iridescent and metallic sheen of the colony surface, that is typical for a *lasR* mutant

^*^LPS and PGN changes in late strains compared to early were described previously exclusively in lineage AA [5].
