# Supplementary material for: Cystic Fibrosis-Niche Adaptation of Pseudomonas aeruginosa Reduces Virulence in Multiple Infection Hosts
Source: PLoS One. 2012 Apr 25;7(4):e35648. doi: 10.1371/journal.pone.0035648 (PMC3338451; doi:10.1371/journal.pone.0035648)
Supplement: Table S3 — Comparison between C57Bl/6NCrl and BALB/cAnCrl infected with P. aeruginosa clonal lineages. (DOC) [file pone.0035648.s004.doc]

**Cystic Fibrosis-niche adaptation of *Pseudomonas aeruginosa* reduces virulence in multiple infection hosts.**

Nicola Ivan Lorè, Cristina Cigana, Ida De Fino, Camilla Riva, Mario Juhas, Stephan Schwager, Leo Eberl, Alessandra Bragonzi.

Online Data Supplement

**Table S3**. Comparison between C57Bl/6NCrl and BALB/cAnCrl infected with *P. aeruginosa* clonal lineages.

| **Mice** | **Strain** | **Dose** | **Mortality %^a^**  **(No. of dead/total mice)** | **LT_50_** |
| --- | --- | --- | --- | --- |
|  |  |  |  |  |
| C57Bl/6NCrl | AA2 | 5x10^6^ | 88,8% (16/18) | 36 h |
| BALB/cAnCrl | AA2 | 5x10^6^ | 53% (9/17) |  |
|  |  |  |  |  |
| C57Bl/6NCrl | AA43 | 5x10^6^ | 0% (0/18) | >96 h |
| BALB/cAnCrl | AA43 | 5x10^6^ | 0% (0/10) | >96 h |
|  |  |  |  |  |
| C57Bl/6NCrl | AA44 | 5x10^6^ | 0% (0/18) | >96 h |
| BALB/cAnCrl | AA44 | 5x10^6^ | 0% (0/9) | >96 h |
|  |  |  |  |  |
|  |  |  |  |  |
| C57Bl/6NCrl | KK1 | 1x10^7^ | 90 % (9/10) | 36 h |
| BALB/cAnCrl | KK1 | 1x10^7^ | 80% (8/10) | 18 h |
|  |  |  |  |  |
| C57Bl/6NCrl | KK2 | 1x10^7^ | 100% (5/5) | 24 h |
| BALB/cAnCrl | KK2 | 1x10^7^ | 70% (7/10) | 24 h |
|  |  |  |  |  |
| C57Bl/6NCrl | KK71 | 1x10^7^ | 0% (0/10) | >96 h |
| BALB/cAnCrl | KK71 | 1x10^7^ | 0% (0/10) | >96 h |
|  |  |  |  |  |
| C57Bl/6NCrl | KK72 | 1x10^7^ | 0% (0/10) | >96 h |
| BALB/cAnCrl | KK72 | 1x10^7^ | 10% (1/10) | >96 h |
